# Community intervention of a single-dose or 2-dose regimen of bivalent human papillomavirus vaccine in schoolgirls in Thailand: vaccine effectiveness 2 years and 4 years after vaccination

**DOI:** 10.1093/jncimonographs/lgae036

**Published:** 2024-11-12

**Authors:** Suchada Jiamsiri, Chulwoo Rhee, Hyeon Seon Ahn, Hyeong-Won Seo, Worrawan Klinsupa, Sunju Park, Jinae Lee, Nakorn Premsri, Chawetsan Namwat, Patummal Silaporn, Jean-Louis Excler, Deok-Ryun Kim, Yun Chon, Joshua N Sampson, Pornjarim Nilyanimit, Sompong Vongpunsawad, Nimesh Poudyal, Lauri E Markowitz, Gitika Panicker, Elizabeth R Unger, Supachai Rerks-Ngarm, Yong Poovorawan, Julia Lynch

**Affiliations:** Department of Disease Control, Ministry of Public Health, Nonthaburi, Thailand; International Vaccine Institute, Seoul, Republic of Korea; International Vaccine Institute, Seoul, Republic of Korea; International Vaccine Institute, Seoul, Republic of Korea; Department of Disease Control, Ministry of Public Health, Nonthaburi, Thailand; International Vaccine Institute, Seoul, Republic of Korea; International Vaccine Institute, Seoul, Republic of Korea; National Vaccine Institute, Nonthaburi, Thailand; Department of Disease Control, Ministry of Public Health, Nonthaburi, Thailand; Department of Disease Control, Ministry of Public Health, Nonthaburi, Thailand; International Vaccine Institute, Seoul, Republic of Korea; International Vaccine Institute, Seoul, Republic of Korea; International Vaccine Institute, Seoul, Republic of Korea; National Cancer Institute, National Institutes of Health, Rockville, MD, USA; Center of Excellence in Clinical Virology, Faculty of Medicine, Chulalongkorn University, Bangkok, Thailand; Center of Excellence in Clinical Virology, Faculty of Medicine, Chulalongkorn University, Bangkok, Thailand; International Vaccine Institute, Seoul, Republic of Korea; Centers for Disease Control and Prevention, Atlanta, GA, USA; Centers for Disease Control and Prevention, Atlanta, GA, USA; Centers for Disease Control and Prevention, Atlanta, GA, USA; Department of Disease Control, Ministry of Public Health, Nonthaburi, Thailand; Center of Excellence in Clinical Virology, Faculty of Medicine, Chulalongkorn University, Bangkok, Thailand; International Vaccine Institute, Seoul, Republic of Korea

## Abstract

**Background:**

With accumulating evidence of single-dose human papillomavirus (HPV) vaccine efficacy in young women, we conducted a community vaccine effectiveness study comparing HPV single-dose and 2-dose regimens (0 and 6 months) of a bivalent HPV vaccine among grade 8 schoolgirls (aged 13-14 years) in Thailand.

**Methods:**

In 2018, eligible grade 8 schoolgirls in Udon Thani (single dose) and Buri Ram (2 doses) provinces were offered HPV vaccine per assigned dose regimen. Concurrently, a cross-sectional survey for measuring baseline HPV prevalence was conducted in grade 10 (n = 2600) and grade 12 unvaccinated schoolgirls (n = 2000) in each province. HPV infection was assessed in first-void urine samples, tested by DNA polymerase chain reaction on the cobas 4800 system (Roche Molecular Diagnostics, Pleasanton, CA). All samples positive on the cobas system and an equal number of negative samples were also tested by Anyplex II HPV28 Detection (Seegene, Seoul, South Korea). The surveys were repeated in 2020 and 2022, when vaccinated grade 8 schoolgirls reached grade 10, and then subsequently grade 12, respectively. Vaccine effectiveness was estimated by comparing the weighted prevalence of HPV-16 or HPV-18 between grade-matched unvaccinated schoolgirls on the baseline survey (2018) and vaccinated schoolgirls in the year-2 (2020) and year-4 (2022) surveys. Adjustment methods were used in the analysis to account for potential differences in sexual behavior due to the noncontemporaneous comparison.

**Results:**

The prevalence of HPV-16 and HPV-18 on the baseline survey among unvaccinated grade 10/grade 12 schoolgirls was 2.90% (95% confidence interval [CI] = 2.54% to 3.31%)/3.98% (95% CI = 3.52% to 4.49%) for Udon Thani and 3.87% (95% CI = 3.46% to 4.34%)/6.13% (95% CI = 5.56% to 6.75%) for Buri Ram. On the year-2 survey, the prevalence among vaccinated grade 10 schoolgirls was 0.57% (95% CI = 0.42% to 0.77%) for Udon Thani and 0.31% (95% CI = 0.21% to 0.47%) for Buri Ram. The 2-year postvaccination crude vaccine effectiveness for the single-dose regimen was estimated at 80.4% (95% CI = 73.9% to 86.9%), and for the 2-dose regimen at 91.9% (95% CI = 88.5% to 95.4%). On the year-4 survey, the prevalence among vaccinated grade 12 schoolgirls was 0.37% (95% CI = 0.25% to 0.56%) for Udon Thani and 0.28% (95% CI = 0.18% to 0.45%) for Buri Ram. Four-year postvaccination crude vaccine effectiveness for the single-dose regimen was estimated at 90.6% (95% CI = 86.6% to 94.6%) and for the 2-dose regimen was estimated at 95.4% (95% CI = 93.2% to 97.6%). All adjustment methods minimally affected vaccine effectiveness for the single-dose and 2-dose regimens. At 4 years after vaccination, the difference in crude vaccine effectiveness between the single-dose and 2-dose regimens was ‒4.79% (95% CI = ‒9.32% to ‒0.25%), meeting the study’s noninferiority criteria.

**Conclusions:**

Our study demonstrated that both single-dose and 2-dose HPV vaccination significantly decreased HPV-16/18 point prevalence 2 years and 4 years after vaccination. Crude vaccine effectiveness at 4 years after vaccination was greater than 90% for both the single-dose and 2-dose regimens; the single-dose regimen was not inferior to the 2-dose regimen. These data show that a single dose of HPV vaccine provides high levels of protection when administered to schoolgirls younger than 15 years of age.

Human papillomavirus (HPV) vaccines are highly effective in preventing HPV infection and sequelae (1). In 2014, the World Health Organization (WHO) Strategic Advisory Group of Experts on Immunization recommended a 2-dose HPV vaccination schedule for female children and adolescents aged 9 to 14 years and a 3-dose regimen for vaccination of female individuals aged 15 years or older (2). The introduction of HPV vaccine has been challenging among low- and middle-income countries (3). Key barriers to HPV vaccine uptake include the target age group, an insufficient vaccine supply, and the cost of the vaccine (4). Subsequently, Thailand first introduced 2-dose HPV vaccination in a pilot province in 2014 (5) that was expanded nationwide through a grade 5 school-based program in 2017.

Thailand’s HPV vaccination program was suspended between 2019 and 2021, however, due to global HPV vaccine supply shortage. In the context of vaccine supply constraints, the WHO Strategic Advisory Group of Experts on Immunization recommended an extended interval between the first and second dose up to 3 to 5 years for countries that had already introduced HPV vaccine among girls aged 9 to 14 years (6). Single-dose HPV vaccination represents a promising strategy to increase vaccine coverage among young girls. Modeling studies suggest that single-dose HPV vaccination may provide substantial public health benefits and be cost-effective (7).

There is growing evidence that a single-dose HPV vaccination regimen can provide high and sustained efficacy against vaccine-type HPV infection (8-10). Existing evidence includes the post hoc analysis of a randomized trial of 3 doses in which not all participants completed the schedule in Costa Rica (8) and the long-term follow-up of a study with an interrupted dose regime in India (9). More recently a randomized controlled trial directly evaluating the efficacy of a single-dose regimen of 2 approved HPV vaccines among female individuals 15 to 20 years of age reported 97.5% vaccine efficacy at 3 years after vaccination for the bivalent HPV vaccine (10). An immuno-bridging study showed that antibody response after a single dose in girls 9 to 14 years of age was comparable to that among vaccine recipients with demonstrated efficacy (11). Consequently, based on the existing evidence, the WHO Strategic Advisory Group of Experts on Immunization endorsed single-dose HPV vaccination for female individuals 9 to 20 years of age (12). No large-scale prospective community intervention study has been conducted, however, to assess vaccine effectiveness in a single-dose HPV vaccine regimen in a real-world setting. Moreover, because HPV epidemiology varies by age group and study setting, evidence generated from Thailand may be helpful in informing HPV immunization policy in other, similar settings. Therefore, we conducted a study to assess the effectiveness of single-dose and 2-dose bivalent HPV vaccination delivered through a school-based immunization program among grade 8 schoolgirls aged 13 to 14 years in Thailand. This article reports the 2-years and 4-years postvaccination results.

## Methods

### Study design and participants

The study protocol was approved by the Thailand Ministry of Public Health Ethical Committee, the International Vaccine Institute Institutional Review Board, and the Chulalongkorn University Faculty of Medicine Ethical Committee. The study is registered at ClinicalTrials.gov with the identifier NCT03747770. The US Centers for Disease Control and Prevention Institutional Review Board deferred to the International Vaccine Institute Institutional Review Board based on the reliance agreement.

The detailed study methodology has been published along with the study protocol (13). In brief, this community vaccine effectiveness study was conducted in 2 provinces, Udon Thani and Buri Ram, starting in December 2018. The 2 northeastern provinces were chosen by the Ministry of Public Health based on their similarities on geographic location, student population, and socioeconomic status. An AS04-adjuvanted HPV-16/18 bivalent HPV vaccine, Cervarix (GlaxoSmithKline, Brentford, UK), was offered to eligible female students by using the existing school-based national immunization program in Thailand. All grade 8 schoolgirls younger than 15 years of age in Udon Thani were offered a single dose, and all grade 8 schoolgirls in Buri Ram were offered 2 doses with a 6-month interval between doses. During the HPV vaccination campaign, 1500 grade 8 schoolgirls in each province were asked about their sexual behavior using a standardized questionnaire to assess sexual activity before HPV vaccination. A subset of 200 eligible grade 8 schoolgirls who had never received an HPV vaccine were recruited for blood sample collection in each province before the HPV vaccination campaign.

The Thai high school system is divided into general high school and vocational school, where grade 10 students can be either general high school-10 or vocational school-1 and grade 12 students can be either general high school-12 or vocational school-3. Sequential cross-sectional surveys of HPV prevalence were administered among schoolgirls in general high school grade 10 or vocational school-1 and general high school grade 12 or vocational school-3 from 2018 through 2022. Each survey included urine collection and a self-administered sexual behavior questionnaire.

In 2018, the first cross-sectional survey, concurrent with the HPV vaccination campaign for grade 8, was conducted to assess the baseline HPV prevalence in the unvaccinated general high school-10/vocational school-1 and general high school-12/vocational school-3 school grade cohorts (n = 2600 for general high school-10/vocational school-1, n = 2000 for general high school-12/vocational school-3 per province). In 2020, when the vaccinated grade 8 cohort became general high school-10/vocational school-1, the second survey (n = 2600 for general high school-10/vocational school-1 per province) was conducted to assess HPV prevalence among the vaccinated cohort 2 years after vaccination. Similarly, when the vaccinated cohort became general high school-12/vocational school-3 schoolgirls in 2022, the third survey (n = 2000 for general high school-2/vocational school-3 per province) was repeated to measure HPV prevalence 4 years after vaccination. During each survey, schoolgirls were systematically selected from school registries in each participating school to obtain representative provincial estimates, with a 1:1 ratio between the 2 school types (ie, 1300 eligible students from each general high school-10 and vocational school-1 in 2018 and 2020 and 1000 from general high school-12 and vocational school-3 in 2018 and 2022). Even though the total number of schoolgirls in general high school are approximately twice the number of girls in vocational school in both provinces, the study oversampled from vocational schools because the schoolgirls in vocational schools were known to have higher sexual activity according to the Ministry of Public Health. In addition, a subset of systematically selected 200 general high school-10/vocational school-1 schoolgirls in 2020 and 200 general high school-12/vocational school-3 schoolgirls in 2022 who received the HPV vaccine in grade 8 per the assigned dose regimen in each province were invited for serologic testing.

### HPV testing and questionnaire data collection

Students collected urine samples at school using a commercially available urine-collection device, Colli-Pee (catalog No. N00176, Novosanis, Ottawa, ON, Canada), containing 7 mL preservative and designed to collect the initial void of urine up to a total volume of 13 mL. The urine samples were transferred under temperature control to the Center of Excellence in Clinical Virology at Chulalongkorn University, where they were processed within 4 days of collection. After centrifuging 10 mL urine, 9 mL of supernatant was discarded, and the pellet was resuspended with residual 1 mL urine. This resuspended pellet was used for testing on the cobas 4800 system (Roche Molecular Diagnostics, Pleasanton, CA; version 2.1.0), a high-throughput, qualitative assay system that detects HPV-16, HPV-18, and 12 other high-risk types of HPV DNA (31, 33, 35, 39, 45, 51, 52, 56, 58, 59, 66, and 68). Residual cobas extracts from any HPV-positive samples and an equal number of adjacent HPV-negative samples were assayed using the Anyplex PV28 Detection (Seegene, Seoul, South Korea) following the manufacturer’s protocol to identify 28 HPV types.

Blood (5 mL) was collected via venipuncture into red-top tubes from eligible participants and allowed to clot at room temperature. Serum was removed and stored at ‒20 °C until shipment on dry ice to the Centers for Disease Control and Prevention laboratory for antibody testing. Sera were tested for immunoglobulin G antibodies against HPV-16, 18, 6, 11, 31, 33, 45, 52, and 58 using the M9ELISA, a direct multiplex enzyme-linked immunosorbent assay with electrochemiluminescence-based detection platform (14). Three serial dilutions were assayed, along with secondary standards, to determine titers using the parallel line method. HPV-16 and HPV-18 results were reported as IU/mL, and the other types were reported as arbitrary units/mL.

The sexual behavior questionnaire was a web-based, self-administered questionnaire with a limited set of questions derived from the Ministry of Public Health national sexual behavioral survey. Enrolled schoolgirls privately accessed the weblink and entered their responses independent of study or school staff using their own cell phone or a hand-held device provided by study staff. No personally identifiable information was collected, and a unique subject identifier was used throughout the study. Only study investigators had access to the password-protected electronic data capture system.

### Statistical analysis

For estimating vaccine effectiveness through the sequential surveys, we excluded participants from analysis who met predefined criteria per our statistical analysis plan. For baseline, the analysis set excluded general high school-10/vocational school-1 and general high school-12/vocational school-3 students enrolled in the baseline survey who would not have met the age criteria for vaccination in grade 8 (eg, would have been younger than 15 years of age in grade 8). For 2-year and 4-year postvaccination surveys, the vaccine effectiveness analysis included schoolgirls enrolled in each survey who had received the HPV vaccine per protocol (ie, single dose for Udon Thani, 2 doses for Buri Ram at 0- and 6-month intervals, with a minimum of 5 months between doses, per Ministry of Public Health policy) in grade 8. Furthermore, we excluded participants who experienced important protocol deviations or had invalid laboratory measurements from the analysis. Details on individuals who were excluded from the vaccine effectiveness analysis are shown in Supplementary Figures 1 through 4 (available online).

We first summarized the demographic characteristics of schoolgirls enrolled in each survey by school grade. Because the ratio of students in general high school and vocational school differed between the provinces and changed over time, we estimated the weighted HPV prevalence using the direct standardization in the cohort of general high school-10/vocational school-1 and general high school-12/vocational school-3 schoolgirls within each province at each time point using the national ratio of 65:35 between general high school and vocational school. We estimated the vaccine effectiveness against HPV-16 or 18 (HPV-16/18), HPV-16 and HPV-18 separately and against HPV-31, 33, or 45 of the single-dose and 2-dose HPV regimen by comparing 2-year and 4-year postvaccination weighted prevalence (ie, general high school-10/vocational school-1 schoolgirls in 2020 and general high school-12/vocational school-3 schoolgirls in 2022) to the baseline weighted prevalence of the respective grade. The primary assessment for each regimen group (single dose and 2 doses) was the unadjusted vaccine effectiveness estimate and 95% confidence interval (CI). The noninferiority comparison between the single-dose and 2-dose regimen was conducted at 4 years after vaccination, with a margin of 10%.

As sensitivity analyses, we calculated weighted HPV prevalence in both provinces by using the observed ratio between general high school and vocational school at the time of each survey and estimated adjusted vaccine effectiveness using 3 different methods. Each method used a different approach to account for the potential differences in sexual behavior or risk between the noncontemporaneous baseline survey respondents (2018), year-2 respondents (2020), and year-4 respondents (2022). The potential for differences in sexual behavior over the period of study were heightened by the occurrence of the COVID-19 pandemic and the institution of public health measures, such as school closures, curfews, and movement restrictions within Thailand. Adjustment method 1 used the prevalence of 7 nonvaccine high-risk HPV types (HPV-35, 39, 56, 58, 59, 66, and 68), with no evidence of cross-protection as a surrogate for sexual activity (15). Adjustment method 2 used a stratified propensity score approach, a technique applied to nonrandomized observational studies that attempts to estimate the effect of an exposure by accounting for the covariates that predict the exposure, with age and the 7 nonvaccine high-risk HPV types as covariates. Adjustment method 3 used the proportion of schoolgirls with self-reported sexual debut from the sexual behavior questionnaire (ie, responding Yes to “Have you ever had sex” question). Details about these adjustment methods are provided in Supplementary Appendix 1 (available online).

We also report the geometric mean titers (GMTs) of antibody with 95% CIs for selected high-risk HPV types before vaccination (grade 8), 2 years after vaccination (general high school-10/vocational school-1), and 4 years after vaccination (general high school-12/vocational school-3). All statistical analyses were conducted using SAS, version 9.4, software (SAS Institute, Cary, NC).

## Results

A total of 7243 and 8215 eligible grade 8 schoolgirls were vaccinated in Udon Thani (single dose) and Buri Ram (2 doses), respectively, per the assigned dose regimen from December 2018 through February 2019 for Udon Thani (single dose) and December 2018 through September 2019 for Buri Ram (2 doses). The per-protocol vaccine coverage was 91.3% in Udon Thani and 91.4% in Buri Ram. The proportion of grade 8 schoolgirls who reported sexual debut at the time of vaccination was slightly higher in Udon Thani than in Buri Ram (7.6%, 95% CI = 6.3% to 9.1%, vs 6.5%, 95% CI = 5.3% to 7.9%), but the difference was not statistically significant.

**Figure 1.**
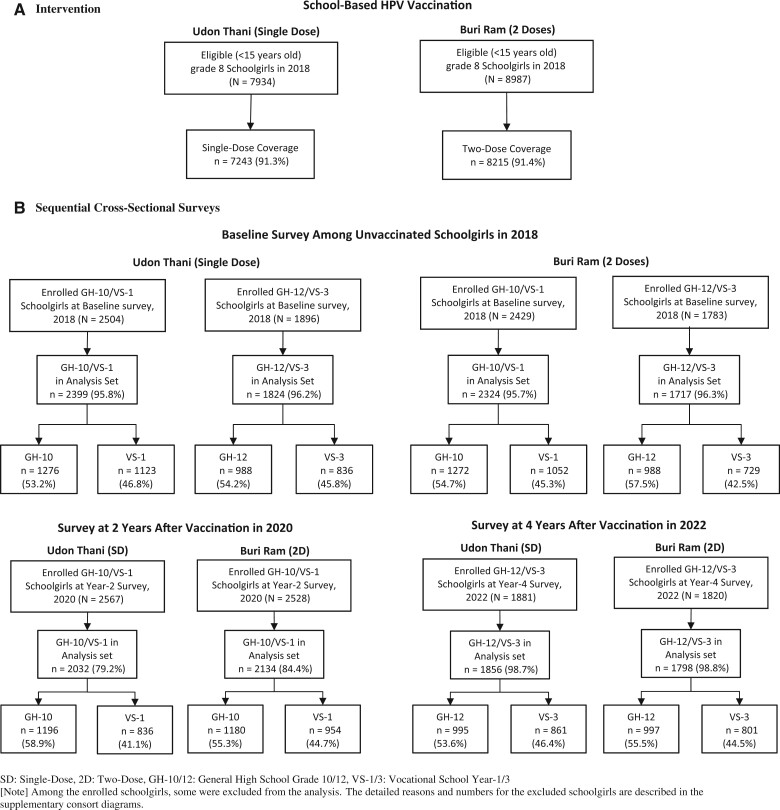
Abbreviated CONSORT diagram of study activities, by year. **A)** Intervention. **B)** Sequential cross-sectional surveys. Among the enrolled schoolgirls, some were excluded from the analysis. The detailed reasons and numbers for the excluded schoolgirls are described in the supplementary CONSORT diagrams. GH-10/12 = general high school-10/12; HPV = human papillomavirus; VS-1/3 = vocational school-1/3.

In the baseline survey, 2504 of 2399 general high school-10/vocational school-1 and 1896 of 1824 general high school-12/vocational school-3 schoolgirls in Udon Thani (single dose) were enrolled and included, and 2429 of 2324 general high school-10/vocational school-1 and 1783 of 1717 general high school-12/vocational school-3 schoolgirls in Buri Ram (2 doses) were enrolled and included, respectively, in the analysis. In the year-2 survey, 2567 schoolgirls in Udon Thani and 2528 schoolgirls in Buri Ram were enrolled, regardless of vaccination history, from general high school-10/vocational school-1. Of those students, 2032 schoolgirls in Udon Thani and 2134 schoolgirls in Buri Ram who had received the HPV vaccine per protocol during grade 8 were included in the analysis (Figure 1). Similarly, on the year-4 survey, 1856 schoolgirls in Udon Thani and 1798 in Buri Ram in general high school-12/vocational school-3 were included. The detailed CONSORT diagram of participants who were enrolled in the 3 sequential surveys are shown by province in Supplementary Figures 1 through 4 (available online), and the demographic characteristics of participants are summarized in Supplementary Table 1 (available online).

In the baseline survey, the weighted prevalence of HPV-16/18 among general high school-10/vocational school-1 schoolgirls in Udon Thani (single dose) and Buri Ram (2 doses) was 2.90% (95% CI = 2.54% to 3.31%) and 3.87% (95% CI = 3.46% to 4.34%), respectively (Table 1). Two years after vaccination, the prevalence of HPV-16/18 among vaccinated general high school-10/vocational school-1 schoolgirls was 0.57% (95% CI = 0.42% to 0.77%) for Udon Thani and 0.31% (95% CI = 0.21% to 0.47%) for Buri Ram. Therefore, the unadjusted estimated 2-year postvaccination vaccine effectiveness for single-dose HPV vaccination against HPV-16/18 was 80.4% (95% CI = 73.9% to 86.9%), and the unadjusted estimated vaccine effectiveness for 2-dose HPV vaccination was 91.9% (95% CI = 88.5% to 95.4%). On the baseline survey, the weighted prevalence of HPV-16/18 among general high school-12/vocational school-3 schoolgirls in Udon Thani (single dose) and Buri Ram (2 doses) was 3.98% (95% CI = 3.52% to 4.49%) and 6.13% (95% CI = 5.56% to 6.75%), respectively. At 4 years after vaccination, the prevalence was 0.37% (95% CI = 0.25% to 0.56%) and 0.28% (95% CI = 0.18% to 0.45%), respectively, among vaccinated schoolgirls. The unadjusted 4-year postvaccination vaccine effectiveness was 90.6% (95% CI = 86.6% to 94.6%) for the single-dose regimen and 95.4% (95% CI = 93.2% to 97.6%) for the 2-dose regimen. Noninferiority criteria were met because the difference in 4-year vaccine effectiveness between the single-dose and 2-dose regimens was ‒4.79% (95% CI = ‒9.32% to ‒0.25%). For both the single-dose and 2-dose regimens, the adjusted vaccine effectiveness estimates changed only slightly using any of the 3 adjustment methods and were within 2 to 3 percentage points of the unadjusted estimates for both methods of calculating provincial-level weighted estimates (Supplementary Table 2, available online).

**Table 1.** Vaccine effectiveness against HPV-16 or HPV-18 2 and 4 years after vaccination, by province and school type

Province	School type	2 years after vaccination							4 years after vaccination						
		**Unvaccinated** ^a^ **(baseline)**			**Vaccinated** ^b^ **(year 2)**			Vaccine effectiveness, %	**Unvaccinated** ^c^ **(baseline)**			**Vaccinated** ^d^ **(year 4)**			Vaccine effectiveness, %
		No.	HPV-16/18	Prevalence, % (95% CI)	No.	HPV-16/18	Prevalence,% (95% CI)		No.	HPV-16/18	Prevalence, % (95% CI)	No.	HPV-16/18	Prevalence, % (95% CI)	
Udon Thani (single dose)	General high school	1276	16	1.25 (0.77 to 2.03)	1196	2	0.17 (0.05 to 0.61)	86.7 (67.1 to 100)	988	21	2.13 (1.39 to 3.23)	995	2	0.20 (0.06 to 0.73)	90.5 (76.9 to 100)
	Vocational school	1123	67	5.97 (4.73 to 7.51)	836	11	1.32 (0.74 to 2.34)	77.9 (64.0 to 91.9)	836	62	7.42 (5.83 to 9.39)	861	6	0.70 (0.32 to 1.51)	90.6 (82.8 to 98.4)
	Total^e^	—		2.90 (2.54 to 3.31)	—		0.57 (0.42 to 0.77)	80.4 (73.9 to 86.9)	—		3.98 (3.52 to 4.49)	—		0.37 (0.25 to 0.56)	90.6 (86.6 to 94.6)
Buri Ram (2 doses)	General high school	1272	25	1.97 (1.33 to 2.89)	1180	1	0.08 (0.01 to 0.48)	95.7 (87.1 to 100)	988	34	3.44 (2.47 to 4.77)	997	1	0.10 (0.02 to 0.57)	97.1 (91.3 to 100)
	Vocational school	1052	78	7.41 (5.98 to 9.16)	954	7	0.73 (0.36 to 1.51)	90.1 (82.5 to 97.7)	729	81	11.1 (9.03 to 13.6)	801	5	0.62 (0.27 to 1.45)	94.4 (89.3 to 99.4)
	Total^e^	—		3.87 (3.46 to 4.34)	—		0.31 (0.21 to 0.47)	91.9 (88.5 to 95.4)	—		6.13 (5.56 to 6.75)	—		0.28 (0.18 to 0.45)	95.4 (93.2 to 97.6)
Difference^f^(95% CI)	—	—		—	—		—	N/A	—		—	—		—	‒4.79 (‒9.32 to ‒0.25)

a Proportion of schoolgirls who had HPV-16 or HPV-18 in general high school grade 10 and vocational school year 1 at the baseline survey. CI = confidence interval; HPV = human papillomavirus; N/A = not applicable.

b Proportion of schoolgirls who had HPV-16 or HPV-18 in general high school grade 10 and vocational school year 1 at the year-2 survey.

c Proportion of schoolgirls who had HPV-16 or HPV-18 in general high school grade 12 and vocational school year 3 at the baseline survey.

d Proportion of schoolgirls who had HPV-16 or HPV-18 in general high school grade 12 and vocational school year 3 at the year-4 survey.

e Weighted total derived by direct standardization (national 65:35 ratio between general high school and vocational school).

f Vaccine effectiveness for the single-dose regimen minus vaccine effectiveness for the 2-dose regimen. The 95% CI was derived using the Wilson method for the prevalence and the Δ method for vaccine effectiveness. For the vaccine effectiveness difference, the 95% CI was derived using the Wald statistic–based confidence interval, with variance estimated by the Δ method.

Vaccine effectiveness estimates for HPV-16 and HPV-18 separately showed some variation by HPV and school type at 2 years after vaccination (Table 2). At 4 years after vaccination, however, all vaccine effectiveness estimates were greater than 90%, except for HPV-18 in vocational school in Udon Thani (single dose). Vaccine effectiveness at 4 years after vaccination was generally higher against HPV-16 than against HPV-18, but 95% CIs overlapped, indicating that differences were not significant.

**Table 2.** Vaccine effectiveness against individual HPV types 16 and 18 2 and 4 years after vaccination, by province and school type

Province	HPV type	School type	2 y after vaccination							4 y after vaccination						
			**Unvaccinated** ^a^ **(baseline)**			**Vaccinated** ^b^ **(year 2)**			Vaccine effectiveness, %	**Unvaccinated** ^c^ **(baseline)**			**Vaccinated** ^d^ **(year 4)**			Vaccine effectiveness, %
			No.	Case No.	Prevalence, % (95% CI)	No.	Case No.	Prevalence, % (95% CI)		No.	Case No.	Prevalence, % (95% CI)	No.	Case, No.	Prevalence, % (95% CI)	
Udon Thani (single dose)	HPV-16	General high school	1276	12	0.94 (0.54 to 1.64)	1196	1	0.08 (0.01 to 0.47)	91.1 (73.0 to 100)	988	13	1.32 (0.77 to 2.24)	995	1	0.10 (0.02 to 0.57)	92.4 (76.8 to 100)
		Vocational school	1123	50	4.45 (3.39 to 5.82)	836	10	1.20 (0.65 to 2.19)	73.1 (55.1 to 91.2)	836	47	5.62 (4.25 to 7.40)	861	3	0.35 (0.12 to 1.02)	93.8 (86.6 to 100)
		Total^e^	—		2.17 (1.86 to 2.53)	—		0.47 (0.34 to 0.66)	78.2 (70.2 to 86.2)	—		2.82 (2.44 to 3.26)	—		0.19 (0.11 to 0.33)	93.4 (89.5 to 97.3)
	HPV-18	General high school	1276	5	0.39 (0.17 to 0.91)	1196	1	0.08 (0.01 to 0.47)	78.7 (32.9 to 100)	988	10	1.01 (0.55 to 1.85)	995	1	0.10 (0.02 to 0.57)	90.1 (69.7 to 100)
		Vocational school	1123	28	2.49 (1.73 to 3.58)	836	1	0.12 (0.02 to 0.67)	95.2 (85.6 to 100)	836	19	2.27 (1.46 to 3.52)	861	3	0.35 (0.12 to 1.02)	84.7 (66.1 to 100)
		Total^e^	—		1.13 (0.91 to 1.40)	—		0.10 (0.05 to 0.20)	91.5 (84.9 to 98.0)	—		1.45 (1.19 to 1.78)	—		0.19 (0.11 to 0.33)	87.1 (79.3 to 94.9)
Buri Ram (2 doses)	HPV-16	General high school	1272	14	1.10 (0.66 to 1.84)	1180	1	0.08 (0.01 to 0.48)	92.3 (76.7 to 100)	988	25	2.53 (1.72 to 3.71)	997	0	0.00 (0.00 to 0.38)	100 (84.8 to 100)
		Vocational school	1052	56	5.32 (4.12 to 6.85)	953	4	0.42 (0.16 to 1.07)	92.1 (84.1 to 100)	729	54	7.41 (5.72 to 9.54)	801	1	0.12 (0.02 to 0.70)	98.3 (95.0 to 100)
		Total^e^	—		2.58 (2.24 to 2.97)	—		0.20 (0.12 to 0.33)	92.2 (88.0 to 96.3)	—		4.24 (3.77 to 4.76)	—		0.04 (0.01 to 0.13)	99.0 (97.7 to 100)
	HPV-18	General high school	1272	12	0.94 (0.54 to 1.64)	1180	0	0.00 (0.00 to 0.32)	100 (65.5 to 100)	988	14	1.42 (0.85 to 2.36)	997	1	0.10 (0.02 to 0.57)	92.9 (78.6 to 100)
		Vocational school	1052	30	2.85 (2.00 to 4.04)	954	4	0.42 (0.16 to 1.07)	85.3 (70.0 to 100)	729	39	5.35 (3.94 to 7.23)	801	4	0.50 (0.19 to 1.28)	90.7 (81.1 to 100)
		Total^e^	—		1.61 (1.35 to 1.93)	—		0.15 (0.08 to 0.26)	90.9 (85.2 to 96.6)	—		2.79 (2.41 to 3.23)	—		0.24 (0.15 to 0.40)	91.4 (86.9 to 95.9)

a Proportion of schoolgirls who had HPV-16 or HPV-18 in general high school grade 10 and vocational school year 1 at the baseline survey. CI = confidence interval; HPV = human papillomavirus.

b Proportion of schoolgirls who had HPV-16 or HPV-18 in general high school grade 10 and vocational school year 1 at the year-2 survey.

c Proportion of schoolgirls who had HPV-16 or HPV-18 positive in general high school grade 12 and vocational school year 3 at the baseline survey.

d Proportion of schoolgirls who had HPV-16 or HPV-18 in general high school grade 12 and vocational school year 3 at the year-4 survey.

e Weighted total derived by direct standardization (national 65:35 ratio between general high school and vocational school). The 95% CI was derived using the Wilson method for the prevalence and the Δ method for vaccine effectiveness. For the vaccine effectiveness difference, the 95% CI was derived using the Wald statistic–based confidence interval, with variance estimated by the Δ method.

We found modest cross-protection against HPV-31, 33, or 45 for both single-dose and 2-dose HPV regimens (Table 3). Unadjusted vaccine effectiveness against these cross-protective high-risk HPV types was 27.3% (95% CI = 8.24% to 46.4%) for Udon Thani (single dose) and 48.2% (95% CI = 32.3% to 64.0%) for Buri Ram (2 doses) at 4 years after vaccination. Vaccine effectiveness at 2 years after vaccination were similar to those 4 years after vaccination for both provinces. For individual cross-protective high-risk HPV types, consistent prevalence reduction was observed for HPV-31, 33, or 45 at 2 years and 4 years after vaccination for both provinces, although some were not statistically significant due to wide 95% CIs. Changes in prevalence observed for other individual, nonvaccine, high-risk HPV types were highly variable among the types (see Supplementary Table 4, available online).

**Table 3.** Vaccine effectiveness against HPV-31, 33, or 45 2 and 4 years after vaccination, by province and school type

Province	School type	2 y after vaccination							4 y after vaccination						
		**Unvaccinated** ^a^ **(baseline)**			**Vaccinated** ^b^ **(year 2)**			Vaccine effectiveness	**Unvaccinated** ^c^ **(baseline)**			**Vaccinated** ^d^ **(year 4)**			Vaccine effectiveness
		No.	Case No.	Prevalence, % (95% CI)	No.	Case No.	Prevalence, % (95% CI)		No.	Case No.	Prevalence, % (95% CI)	No.	Case No.	Prevalence, % (95% CI)	
Udon Thani (single dose)	General high school	1276	8	0.63 (0.32 to 1.23)	1196	4	0.33 (0.13 to 0.86)	46.7 (‒17.0 to 100)	988	15	1.52 (0.92 to 2.49)	995	15	1.51 (0.92 to 2.47)	0.70 (‒70 to 71.2)
	Vocational school	1123	18	1.60 (1.02 to 2.52)	836	12	1.44 (0.82 to 2.49)	10.4 (‒54.0 to 75.4)	836	26	3.11 (2.13 to 4.52)	861	13	1.51 (0.88 to 2.57)	51.5 (19.5 to 83.4)
	Total^e^	—		0.97 (0.77 to 1.22)	—		0.72 (0.55 to 0.94)	25.7 (‒0.61 to 52.0)	—		2.08 (1.75 to 2.46)	—		1.51 (1.24 to 1.84)	27.3 (8.24 to 46.4)
Buri Ram (2 doses)	General high school	1272	7	0.55 (0.27 to 1.13)	1180	6	0.51 (0.23 to 1.10)	7.60 (‒93.0 to 100)	988	12	1.21 (0.70 to 2.11)	997	5	0.50 (0.21 to 1.17)	58.7 (15.8 to 100)
	Vocational school	1052	24	2.28 (1.54 to 3.37)	954	7	0.73 (0.36 to 1.51)	67.8 (40.9 to 94.8)	729	23	3.16 (2.11 to 4.69)	801	15	1.87 (1.14 to 3.07)	40.6 (2.50 to 78.8)
	Total^e^	—		1.16 (0.94 to 1.43)	—		0.59 (0.44 to 0.79)	49.2 (30.7 to 67.7)	—		1.89 (1.58 to 2.26)	—		0.98 (0.77 to 1.26)	48.2 (32.3 to 64.0)

a Proportion of schoolgirls who had HPV-31, 33, or 45 in general high school grade 10 and vocational school year 1 at the baseline survey. CI = confidence interval; HPV = human papillomavirus.

b Proportion of schoolgirls who had HPV-31, 33, or 45 in general high school grade 10 and vocational school year 1 at the year-2 survey.

c Proportion of schoolgirls who had HPV-31, 33, or 45 in general high school grade 12 and vocational school year 3 at the baseline survey.

d Proportion of schoolgirls who had HPV-31, 33, or 45 in general high school grade 12 and vocational school year 3 at the year-4 survey.

e Weighted total derived by direct standardization (national 65:35 ratio between general high school and vocational school). The 95% CI was derived using the Wilson method for the prevalence and the Δ method for the vaccine effectiveness. For the vaccine effectiveness difference, the 95% CI was derived using the Wald statistic–based confidence interval, with variance estimated by the Δ method.

**Figure 2.**
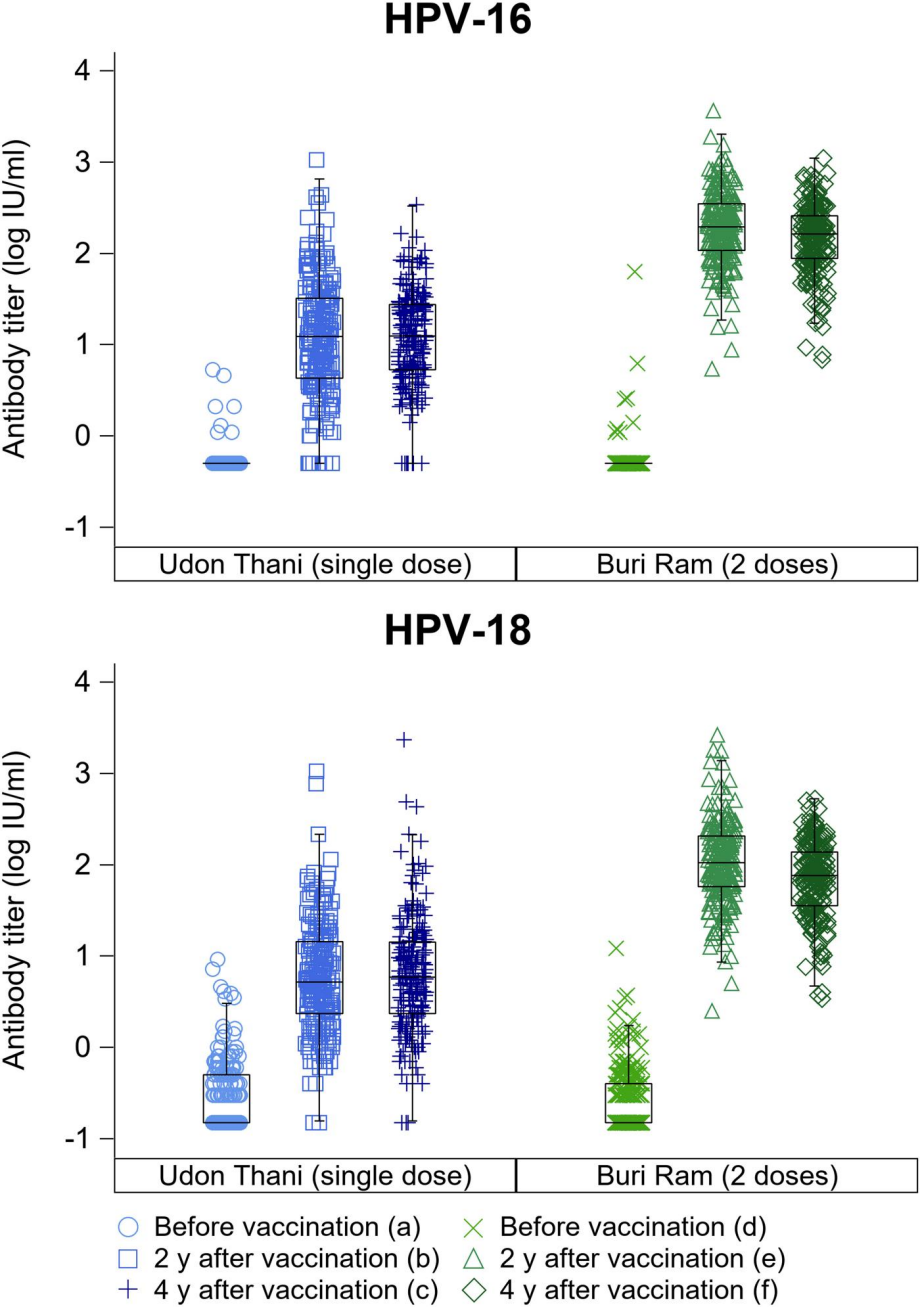
HPV-16 and HPV-18 antibody titers in grade 8 schoolgirls at baseline, grade 10 schoolgirls at 2 years after vaccination, and grade 12 schoolgirls 4 years after vaccination. Results for HPV types are reported in IU. Samples with undetectable titers were replaced by multiplying the type-specific lower limit of quantitation by 0.5. ^a,d^ Grade 8 schoolgirls before the HPV vaccination campaign in Udon Thani (a) and Buri Ram (d). ^b,e^ Two-year postvaccination findings among general high school-10 and vocational school-1 in Udon Thani (b) and Buri Ram (e). ^c,f^ Four-year postvaccination among general high school-12 and vocational school-3 in Udon Thani (c) and Buri Ram (f). HPV = human papillomavirus.

Figure 2 shows the distribution of HPV-16 and HPV-18 GMTs for grade 8 schoolgirls before HPV vaccination and for general high school-10/vocational school-1 schoolgirls and general high school-12/vocational school-3 schoolgirls 2 and 4 years after vaccination respectively. The HPV-16 GMT increased from 0.53 IU/mL (95% CI = 0.51 to 0.55) before vaccination to 12.2 IU/mL (95% CI = 9.93 to 14.9)/11.7 IU/mL (95% CI = 9.89 to 13.9) at 2 years and 4 years, respectively, after vaccination in Udon Thani (single dose) and from 0.54 IU/mL (95% CI = 0.51 to 0.57) to 192 IU/mL (95% CI = 168 to 219)/145 IU/mL (95% CI = 128 to 163), respectively, in Buri Ram (2 doses). Similarly, the HPV-18 GMT increased from 0.28 IU/mL (95% CI = 0.25 to 0.32) to 5.85 IU/mL (95% CI = 4.82 to 7.09)/6.16 IU/mL (95% CI = 5.03 to 7.55), respectively, in Udon Thani (single dose) and from 0.27 IU/mL (95% CI = 0.24 to 0.30) to 105 IU/mL (95% CI = 90.9 to 122)/69.1 IU/mL (95% CI = 60.6 to 78.8), respectively, in Buri Ram (2 doses). HPV-31, 33, and 45 GMTs by province are shown in Supplementary Figure 5 (available online).

## Discussion

This study is the first to report bivalent HPV vaccine effectiveness of single-dose and 2-dose regimens through a community intervention design. We evaluated the prevalence of HPV-16/18 at 2 and 4 years after vaccination among grade 8 schoolgirls younger than 15 years of age. With both regimens, the prevalence of HPV-16/18 was significantly lower than with unvaccinated historic controls of the same grade.

Our 2-year and 4-year postvaccination vaccine effectiveness point estimates for the single-dose regimen (80.4% and 90.6%) using single-time-point prevalent infection are similar to prior studies assessing single-dose HPV vaccine effectiveness with a similar endpoint. In a post hoc analysis of 2 bivalent HPV vaccine trials, the authors reported a single-dose vaccine efficacy of 85.7% (95% CI = 70.7% to 93.7%) against single-time-point detection of HPV-16/18 among women who were negative for vaccine-type HPV at the time of vaccination (16). Similar vaccine effectiveness was found in a study in Mongolia that evaluated prevalent HPV-16/18 detection 6 years after single-dose quadrivalent HPV vaccination. Among adolescents aged 11 to 17 years, the prevalence of HPV-16/18 was 92% (95% CI = 44% to 99%) lower than in an unvaccinated group (17).

In HPV vaccine clinical trials, persistent HPV infection, defined as detection of HPV DNA in 2 consecutive cervical swabs at a 6-month intervals, has been a widely used endpoint (18). As single-time-point detection cannot discriminate between persistent and transient infection or recent deposition from a sexual partner rather than a true infection (19,20), persistent infection is a more rigorous endpoint. Persistent infection is associated with risk of progression to HPV-related high-grade cervical disease and considered a surrogate for a clinical endpoint (21). Several recently reported trials that used a persistent infection endpoint have demonstrated a higher vaccine effectiveness for single-dose regimens than reported here. An observational study in India that followed a cohort of quadrivalent HPV vaccine recipients from a suspended phase 3 clinical trial reported a uniformly low incidence of persistent HPV-16/18 infection among participants who received 1, 2, or 3 doses of vaccine. Among these women, vaccinated at ages 10 to 18 years and before marriage, the vaccine effectiveness 10 years after vaccination for the single-dose regimen was 95.4% (95% CI = 85.0% to 99.9%) (9). A randomized clinical trial in Kenya that assessed the efficacy of 2 HPV vaccines 36 months after vaccination among women who were negative for vaccine-type HPV at enrollment found vaccine efficacy against persistent HPV-16/18 infection of 97.5% (95% CI = 90.0% to 99.4%) for bivalent HPV vaccine (10). The difference in the endpoints used, single-time-point detection vs persistent infection, and the number of participants with preexisting infection at the time of vaccination likely contributed to the relatively lower vaccine effectiveness of the single-dose regimen assessed in our study.

Because we aimed to measure real-world vaccine effectiveness, schoolgirls were not tested for preexisting HPV-16/18 infection or HPV serostatus at the time of vaccination, in contrast to previous clinical trials that assessed efficacy of HPV vaccine after excluding participants based on baseline HPV DNA testing or serostatus (10,16). Based on the self-reported sexual behavior questionnaire, at least 7.6% of grade 8 schoolgirls in Udon Thani (single dose) and 6.5% in Buri Ram (2 doses) were already sexually active before vaccination, and it is likely that some had already acquired HPV-16 or HPV-18. We note the relatively lower vaccine effectiveness 2 years after vaccination, especially against HPV-16, the most common HPV type among schoolgirls in Thailand (22). Considering the likelihood of preexisting infections, however, particularly among vocational school students, who have higher rates of sexual activity, we think that this should be interpreted cautiously. Although we were unable to clearly differentiate preexisting infections from break-through infections, the observed increase in vaccine effectiveness from 2 years to 4 years after vaccination, particularly in vocational school, against HPV-16, suggests the gradual clearance of preexisting and recently acquired infections over the study period. The higher proportion of schoolgirls with self-reported sexual debut in Udon Thani may have resulted in a higher number with preexisting infection before vaccination, which resulted in a larger increase in vaccine effectiveness observed in Udon Thani (single dose). We believe that vaccine effectiveness 4 years after vaccination better represents the true protection conferred by the HPV vaccine, and our findings further support the WHO recommendation on HPV single-dose vaccination for female individuals 9 to 20 years of age (12).

We explored the effect of single-dose and 2-dose vaccination on the prevalence of other high-risk HPV types with prior evidence of cross-protection from the bivalent vaccine and found a modest prevalence reduction in both the single-dose (27.3%) and 2-dose (48.2%) provinces 4 years after vaccination. Although bivalent HPV vaccine may possibly provide partial cross-protection against HPV types other than HPV-31, 33, and 45, as well, we excluded these types in a composite endpoint because cross-protection against these types has not been frequently reported (23,24). A follow-up analysis of the Costa Rica Vaccine trial demonstrated statistically significant cross-protection against a composite outcome of HPV-31, 33, and 45, with a vaccine effectiveness of 54.4% (95% CI = 21.0% to 73.7%) for single-dose bivalent HPV vaccination (25). The 10-year follow-up study in India demonstrated a significant vaccine effectiveness of 43.5% (95% CI = 25.4% to 56.5%) against HPV-31, 33, and 45 after single-dose quadrivalent HPV vaccination (9). As cross-protection was reported to be largely driven by vaccine effectiveness against HPV-31 and HPV-45 (24), our selection of composite cross-protection endpoint may have underestimated the level of cross-protection. Nevertheless, even this modest effect may have important clinical implications at a population level for reducing other high-risk HPV types that can lead to prevention of cancerous lesions.

Prior clinical studies have demonstrated the kinetics of antibody responses to HPV vaccination, with an early rise after the initial series (26) and a subsequent persistent plateau level of antibody, now reported up to 11 years after vaccination for 1, 2, or 3 doses (8). Although all regimens demonstrate the same pattern over time, several studies, including ours, show that a single-dose regimen consistently results in lower GMTs than a multidose regimen. Even though the HPV-16 antibody level in the single-dose group was reported to be 4- to 10-fold lower than that of a multiple-dose regimen, the sustained antibody level is substantially higher than the immune response after natural infection (27,28). It will be important to continue documenting the duration of the antibody plateau and protection that single-dose HPV vaccination provides over longer periods and in varied populations and settings.

A major limitation of this study is the potential imbalance between the unvaccinated general high school-10/vocational school-1 and general high school-12/vocational school-3 schoolgirls enrolled in the baseline survey and vaccinated schoolgirls of the same grade enrolled in the year-2 and year-4 surveys. In using a historical comparison group, we assumed that student behavior related to the risk of HPV acquisition would not change over the 4-year interval, but during this interval, Thailand and the world experienced the COVID-19 pandemic, which resulted in school closures and various other restrictions on social interactions, especially around the year-2 survey in 2020. The Thai government implemented pandemic control measures nationally and concurrently in both provinces. As these social restrictions had the potential to affect student sexual behavior, we used 3 adjustment methods to assess and correct for differences in sexual behavior and risk for HPV acquisition between noncontemporaneous schoolgirls of the same grade. All adjustments minimally changed the 2-year and 4-year postvaccination vaccine effectiveness estimates. Other potential imbalances may exist between the students enrolled in the 2 separate cross-sectional surveys that could affect our estimates, including differences in demographic factors. We did not see any statistical differences, however, in HPV-16/18 prevalence between residential districts, and we calculated vaccine effectiveness based on school type weighted prevalence to be representative of the province school enrollments. Our vaccine effectiveness analyses were restricted to individuals who received HPV vaccination at baseline, and we were unable to estimate vaccine effectiveness for the entire grade cohort nor assess herd protection. It must be noted that students enrolled in the year-2 and year-4 surveys and in the vaccine effectiveness assessment had voluntarily agreed to vaccination 2 years and 4 years earlier and completed their vaccination according to protocol. Although we attempted to balance the age of the vaccinated group in the 2-year and 4-year postvaccination surveys and the baseline survey comparison group by creating age boundaries, there may be other differences between the groups that introduce bias. Willingness to be vaccinated and completion of the vaccination activities may be associated with behaviors that are linked with the risk of HPV acquisition. Finally, HPV infections among vaccinated students could represent vaccine failure or preexisting infections before vaccination. Because we did not assess HPV prevalence at grade 8, we do not know whether the HPV prevalence was similar between the 2 provinces at the time of the vaccination campaign.

In this study, we report that the single-dose HPV vaccine administered to grade 8 schoolgirls younger than 15 years of age confers high vaccine effectiveness, estimated at 4 years after vaccination in a community intervention study in Thailand. The single-dose regimen offers the potential to expand vaccine coverage by reducing delivery challenges and cost.

## Supplementary Material

lgae036_Supplementary_Data

## Data Availability

Individual participant data that underlie the results reported in this article, after deidentification (text, tables, figures, and appendices) and including data dictionaries and analytic code, will be publicly available following completion of the study. Data can be found at https://doi.org/10.6084/m9.figshare.26240798.
