# Supplementary material for: Community intervention of a single-dose or 2-dose regimen of bivalent human papillomavirus vaccine in schoolgirls in Thailand: vaccine effectiveness 2 years and 4 years after vaccination
Source: J Natl Cancer Inst Monogr. 2024 Nov 12;2024(67):346–57. doi: 10.1093/jncimonographs/lgae036 (PMC11555278; doi:10.1093/jncimonographs/lgae036)
Supplement: lgae036_Supplementary_Data [file lgae036_supplementary_data.docx]

**Supplementary Table 1. Demographics of Schoolgirls Included in Two- and Four-Year Post-Vaccination Vaccine Effectiveness Analysis Set**

| **Variable** | **Two-Year Post-Vaccination Analysis Set** | | | | **Four-Year Post-Vaccination Analysis Set** | | | |
| --- | --- | --- | --- | --- | --- | --- | --- | --- |
|  | **Udon Thani (SD)** | | **Buri Ram (2D)** | | **Udon Thani (SD)** | | **Buri Ram (2D)** | |
|  | **Baseline Survey**  **N=2,399** | **Year-2 Survey**  **N=2,032** | **Baseline Survey**  **N=2,324** | **Year-2 Survey**  **N=2,134** | **Baseline Survey N=1,824** | **Year-4 Survey N=1,856** | **Baseline Survey N=1,717** | **Year-4 Survey N=1,798** |
| **Residential District** | | | | | | | | |
| Central District | 1,265 (52.7%) | 972 (47.8%) | 736 (31.7%) | 657 (30.8%) | 857 (47.0%) | 979 (52.7%) | 567 (33.0%) | 494 (27.5%) |
| Rural Districts | 1,134 (47.3%) | 1,060 (52.2%) | 1,588 (68.3%) | 1,477 (69.2%) | 967 (53.0%) | 877 (47.3%) | 1,150 (67.0%) | 1,304 (72.5%) |
| **School Type** | | | | | | | | |
| General High School | 1,276 (53.2%) | 1,196 (58.9%) | 1,272 (54.7%) | 1,180 (55.3%) | 988 (54.2%) | 995 (53.6%) | 988 (57.5%) | 997 (55.5%) |
| Vocational School | 1,123 (46.8%) | 836 (41.1%) | 1,052 (45.3%) | 954 (44.7%) | 836 (45.8%) | 861 (46.4%) | 729 (42.5%) | 801 (44.5%) |
| **Age at Survey** | | | | | | | | |
| Mean ± Stdev | 16.4 (0.38) | 16.3 (0.36) | 16.3 (0.37) | 16.2 (0.34) | 18.4 (0.38) | 18.2 (0.36) | 18.3 (0.37) | 18.0 (0.33) |
| Median  (Q1, Q3) | 16.4  (16.2, 16.7) | 16.3  (16.1, 16.6) | 16.3  (16.0, 16.5) | 16.2  (15.9, 16.4) | 18.4  (18.2, 18.7) | 18.2  (18.0, 18.4) | 18.3  (18.0, 18.5) | 18.0  (17.8, 18.3) |
| Min - Max | 14.6-17.1 | 15.0-17.0 | 14.8-17.1 | 15.0-17.0 | 16.4-19.1 | 16.9-18.9 | 16.6-19.1 | 17.1-18.8 |

Stdev: Standard deviation, SD: Single-Dose, 2D: Two-Dose

**Supplementary Table 2. Adjusted Vaccine Effectiveness at Two- and Four-Year Post-Vaccination**

| **Method for weighted total** | **Province** | **Two-Year Post-Vaccination VE** | | | | **Four-Year Post-Vaccination VE** | | | |
| --- | --- | --- | --- | --- | --- | --- | --- | --- | --- |
|  |  | **Unadjusted** | **Adjusted ^a^ (95% CI)** | **Adjusted ^b^ (95% CI)** | **Adjusted ^c^ (95% CI)** | **Unadjusted** | **Adjusted ^a^ (95% CI)** | **Adjusted ^b^ (95% CI)** | **Adjusted ^c^ (95% CI)** |
| Direct  Standardization^d^ | Udon Thani  (SD) | 80.4  (73.9, 86.9) | 82.3  (76.1, 88.6) | 80.7  (74.4, 87.1) | 78.4  (71.2, 85.6) | 90.6  (86.6, 94.6) | 91.6  (87.9, 95.2) | 91.1  (87.4, 94.9) | 90.7  (86.7, 94.6) |
|  | Buri Ram  (2D) | 91.9  (88.5, 95.4) | 90.4  (86.2, 94.6) | 91.7  (88.2, 95.3) | 91.3  (87.6, 95.0) | 95.4  (93.2, 97.6) | 94.8  (92.2, 97.3) | 95.0  (92.7, 97.3) | 95.0  (92.6, 97.4) |
| Horvitz-Thompson^e^ | Udon Thani  (SD) | 83.0 (76.4, 89.6) | 83.1 (76.2, 90.1) | 83.2  (76.7, 89.7) | 80.2 (72.4, 88.0) | 90.8  (86.3, 95.3) | 91.7 (87.5, 95.8) | 91.4 (87.2, 95.7) | 90.8 (86.3, 95.3) |
|  | Buri Ram  (2D) | 93.6 (90.5, 96.8) | 91.4 (87.0, 95.8) | 93.6  (90.3, 96.8) | 92.4 (88.6, 96.2) | 96.1  (93.9, 98.4) | 95.2 (92.4, 98.0) | 96.0 (93.7, 98.2) | 95.7 (93.1, 98.2) |

SD: Single-Dose, 2D: Two-Dose, VE: Vaccine Effectiveness

^a^ Adjusted using non-targeted 7 types (35, 39, 56, 58, 59, 66, 68)

^b^ Adjusted using propensity score method

^c^ Adjusted using self-reported sexual activity

^d^ Weighted total derived by direct standardization (National 65:35 ratio between General High School and Vocational School)

^e^ Weighted total derived by Horvitz-Thompson method (Observed ratio between General High School and Vocational School at the time of each survey)

95% confidence interval was derived using the Delta Method for all VEs

**Supplementary Table 3. Prevalence and Percent Reduction of High-Risk Human Papillomavirus (HPV) Types between Baseline and Year-2 Cross-Sectional Surveys**

| **Cross-Protection** | **HPV Type** | **Udon Thani (SD)** | | | **Buri Ram (2D)** | | |
| --- | --- | --- | --- | --- | --- | --- | --- |
|  |  | **Prevalence in Unvaccinated^a^ (%)** | **Prevalence in Vaccinated^b^ (%)** | **Prevalence reduction**  **(95% CI)** | **Prevalence in Unvaccinated^a^ (%)** | **Prevalence in Vaccinated^b^ (%)** | **Prevalence reduction**  **(95% CI)** |
| **Consistent Evidence of Cross-Protection** | **HPV 31** | 0.37 | 0.31 | 17.6  (-28, 63.5) | 0.43 | 0.09 | 78.9  (61.4, 96.4) |
|  | **HPV 33** | 0.34 | 0.23 | 31.1  (-11, 73.3) | 0.57 | 0.44 | 22.8  (-12, 58.1) |
|  | **HPV 45** | 0.26 | 0.18 | 30.2  (-19, 79.1) | 0.22 | 0.06 | 74.7  (47.1, 100) |
| **Non-consistent Evidence of Cross-Protection** | **HPV 35** | 0.00 | 0.04 | - | 0.22 | 0.22 | -1.3  (-71, 68.5) |
|  | **HPV 39** | 0.68 | 0.69 | -1.6 (-41, 37.9) | 0.79 | 0.83 | -4.8 (-42, 32.7) |
|  | **HPV 51** | 1.25 | 0.91 | 27.0  (4.12, 49.8) | 1.40 | 1.56 | -12  (-41, 17.8) |
|  | **HPV 52** | 1.52 | 1.38 | 8.86  (-15, 33.2) | 1.84 | 1.45 | 21.4  (1.64, 41.2) |
|  | **HPV 56** | 0.79 | 1.05 | -32 (-77, 12.3) | 1.11 | 1.05 | 5.57 (-24, 34.8) |
|  | **HPV 58** | 1.52 | 1.73 | -14 (-43, 14.8) | 2.16 | 1.10 | 49.1 (35.5, 62.6) |
|  | **HPV 59** | 1.28 | 1.09 | 14.7 (-11, 39.9) | 1.53 | 1.41 | 7.76 (-17, 32.1) |
|  | **HPV 66** | 1.49 | 1.75 | -17 (-47, 12.5) | 1.66 | 1.36 | 18.2 (-3.2, 39.7) |
|  | **HPV 68** | 0.57 | 0.68 | -19 (-68, 29.6) | 0.67 | 0.86 | -28 (-76, 19.2) |

SD: Single-Dose, 2D: Two-Dose

^a^ Proportion of Schoolgirls who are positive for corresponding HPV type in General High School Grade 10 and Vocational School Year-1 at Baseline survey

^b^ Proportion of Schoolgirls who are positive for corresponding HPV type in General High School Grade 10 and Vocational School Year-1 at Year-2 survey

Prevalences shown in this table are weighted estimates derived by direct standardization (National 65:35 ratio between General High School and Vocational School)

95% confidence interval was derived using the Delta Method

**Supplementary Table 4. Prevalence and Percent Reduction of High-Risk Human Papillomavirus (HPV) Types between Baseline and Year-4 Cross-Sectional Surveys**

| **Cross-Protection** | **HPV Type** | **Udon Thani (SD)** | | | **Buri Ram (2D)** | | |
| --- | --- | --- | --- | --- | --- | --- | --- |
|  |  | **Prevalence in Unvaccinated^a^ (%)** | **Prevalence in Vaccinated^b^ (%)** | **Prevalence reduction**  **(95% CI)** | **Prevalence in Unvaccinated^a^ (%)** | **Prevalence in Vaccinated^b^ (%)** | **Prevalence reduction**  **(95% CI)** |
| **Consistent evidence of Cross-Protection** | **HPV 31** | 1.05 | 0.78 | 25.2  (-2.3, 52.8) | 0.65 | 0.26 | 59.6  (36.5, 82.7) |
|  | **HPV 33** | 0.77 | 0.60 | 22.9  (-10, 55.7) | 0.86 | 0.72 | 16.0  (-17, 49.1) |
|  | **HPV 45** | 0.26 | 0.13 | 49.2  (6.51, 91.9) | 0.46 | 0.00 | 100  (86.5, 100) |
| **Non-consistent Evidence of Cross-Protection** | **HPV 35** | 0.08 | 0.13 | -56  (-227, 100) | 0.25 | 0.37 | -51 (-148, 46.0) |
|  | **HPV 39** | 1.20 | 1.48 | -24 (-61, 13.5) | 1.96 | 1.31 | 33.2 (14.7, 51.7) |
|  | **HPV 51** | 1.87 | 2.90 | -55  (-90, -19) | 3.22 | 1.61 | 50.0  (38.1, 61.8) |
|  | **HPV 52** | 2.74 | 2.43 | 11.4  (-7.7, 30.5) | 2.62 | 2.20 | 15.9  (-2.9, 34.7) |
|  | **HPV 56** | 1.84 | 2.01 | -9.6 (-37, 17.8) | 2.09 | 2.33 | -12 (-38, 14.5) |
|  | **HPV 58** | 2.65 | 3.13 | -18 (-42, 5.96) | 3.58 | 2.94 | 17.8 (2.11, 33.6) |
|  | **HPV 59** | 1.70 | 1.96 | -16 (-45, 14.1) | 1.68 | 1.94 | -16 (-45, 14.3) |
|  | **HPV 66** | 2.17 | 2.17 | -0.04 (-24, 23.5) | 2.28 | 1.66 | 27.2 (9.02, 45.4) |
|  | **HPV 68** | 1.09 | 1.08 | 1.17 (-32, 34.2) | 1.31 | 1.05 | 20.2 (-5.5, 45.9) |

SD: Single-Dose, 2D: Two-Dose

^a^ Proportion of Schoolgirls who are positive for corresponding HPV type in General High School Grade 12 and Vocational School Year-3 at baseline survey

^b^ Proportion of Schoolgirls who are positive for corresponding HPV type in General High School Grade 12 and Vocational School Year-3 at Year-4 survey

Prevalences shown in this table are weighted estimates derived by direct standardization (National 65:35 ratio between General High School and Vocational School)

95% confidence interval was derived using the Delta Method

**Supplementary Figure 1. Consort Diagram of Grade 8 Human Papillomavirus (HPV) Vaccination and Per-Protocol Coverage by Province**

SD: Single-Dose, 2D: Two-dose, PP: Per-Protocol (Single-Dose for Udon Thani, Two-Dose for Buri Ram)

Grade 8 Schoolgirls <15 years old in 2018

(N=7,970)

Received 1^st^ dose

(n=7,243)

**PP Coverage: 91.3%**

Received 1^st^  dose

(n=8,494)

279 Participants excluded

- Missed 2^nd^ dose (n=278)

- Did not receive HPV vaccine per-protocol (n=1)

691 Participants excluded

- Did not agree to HPV vaccination (n=690)

- Did not receive HPV vaccine per-protocol (n=1)

Eligible Schoolgirls (n=7,934)

Grade 8 Schoolgirls <15 years old in 2018

(N=9,022)

35 Non-eligible Schoolgirls

- Health condition (n=4)

- Pregnant (n=3)

- History of prior HPV vaccine (n=28)

493 Participants excluded

- Did not agree to HPV vaccination (n=493)

Eligible Schoolgirls (n=8,987)

Received 2^nd^ dose

(n=8,215)

**PP Coverage: 91.4%**

**Udon Thani (SD)**

**Buri Ram (2D)**

36 Non-eligible Schoolgirls

- Health condition (n=7)

- Pregnant (n=4)

- History of prior HPV vaccination (n=25)

**Supplementary Figure 2. Consort Diagram of Baseline Cross-Sectional Survey for Vaccine Effective Analysis Set**

1. **Grade 12**
2. **Grade 10**

Enrolled GH-10/VS-1 Schoolgirls at Baseline survey, 2018 (N=2,504)

Schoolgirls available for analysis (n=2,489)

Schoolgirls available for analysis (n=2,425)

15 Participants excluded

- No consent/assent (n=6)

- No urine sample (n=4)

- Urine sample < 10mL (n=4)

- Invalid lab result (n=1)

Enrolled GH-10/VS-1 Schoolgirls at Baseline survey, 2018 (N=2,429)

4 Participants excluded

- No consent/assent (n=1)

- No urine sample (n=1)

- Urine sample < 10mL (n=1)

- Invalid lab result (n=1)

**Udon Thani (SD)**

**Buri Ram (2D)**

90 Participants excluded

- Out of age boundary for VE set* (n=90)

Schoolgirls included in analysis set (n=2,399)

101 Participants excluded

- Out of age boundary for VE set* (n=101)

Schoolgirls included in analysis set (n=2,324)

Enrolled GH-12/VS-3 Schoolgirls at Baseline survey, 2018 (N=1,896)

Schoolgirls available for analysis (n=1,887)

Schoolgirls available for analysis (n=1,773)

9 Participants excluded

- No consent/assent (n=1)

- No urine sample (n=3)

- Urine sample < 10mL (n=2)

- Urine sample without proper label (n=2)

- Invalid lab result (n=1)

Enrolled GH-12/VS-3 Schoolgirls at Baseline survey, 2018 (N=1,783)

10 Participants excluded

- No consent/assent (n=3)

- No urine sample (n=4)

- Urine sample < 10mL (n=1)

- Invalid lab result (n=2)

63 Participants excluded

- Out of age boundary for VE set* (n=63)

Schoolgirls included in analysis set (n=1,824)

56 Participants excluded

- Out of age boundary for VE set* (n=56)

Schoolgirls included in analysis set (n=1,717)

* Schoolgirls who would have been age < 15 years old in Grade 8 based on date of birth

SD: Single-Dose, 2D: Two-Dose, GH-10: General High School Grade 10, VS-1: Vocational School Year-1, GH-12: General High School Grade 12, VS-3: Vocational School Year-3

**Supplementary Figure 3. Consort Diagram of Two-Year Post-Vaccination Survey for Vaccine Effective Analysis Set**

Enrolled GH-10/VS-1 Schoolgirls at Year-2 survey, 2020 (N=2,567)

Schoolgirls available for analysis (n=2,539)

Schoolgirls available for analysis (n=2,478)

28 Participants excluded

- No urine sample (n=11)

- Not eligible to enroll (n=2)

- Invalid lab result (n=15)

Enrolled GH-10/VS-1 Schoolgirls at Year-2 survey, 2020 (N=2,528)

50 Participants excluded

- Consent after study activity (n=1)

- Not eligible to enroll (n=3)

- Urine <10 ml (n=3)

- Missing consent form (n=29)

- Invalid lab results (n=14)

**Udon Thani (SD)**

**Buri Ram (2D)**

507 Participants excluded

- Non-vaccinated at baseline (n=507)

Schoolgirls included in analysis set (n=2,032)

344 Participants excluded

- Non-vaccinated at baseline (n=344)

Schoolgirls included in analysis set (n=2,134)

SD: Single-Dose, 2D: Two-Dose, GH-10: General High School Grade 10, VS-1: Vocational School Year-1, VES: Vaccine Effectiveness Set

**Supplementary Figure 4. Consort Diagram of Four-Year Post-Vaccination Survey for Vaccine Effective Analysis Set**

Enrolled GH-12/VS-3 Schoolgirls at Year-4 survey, 2022 (N=1,881)

Schoolgirls available for analysis and included in analysis set* (n=1,856)

Schoolgirls available for analysis and included in analysis set* (n=1,798)

25 Participants Excluded

- No urine sample (n=4)

- No urine sample and missing consent (n=5)

- Not eligible to enroll (n=12)

- Invalid lab result (n=4)

Enrolled GH-12/VS-3 Schoolgirls at Year-4 survey, 2022 (N=1,820)

22 Participants Excluded

- Collected urine sample before consent (n=4)

- No urine sample (n=5)

- Not eligible to enroll (n=9)

- Urine less than 10ml (n=1)

- Invalid lab result (n=3)

--

**Udon Thani (SD)**

**Buri Ram (2D)**

SD: Single Dose, 2D: Two-Dose, GH-12: General High School Grade 12, VS-3: Vocational School Year-3, VES: Vaccine Effectiveness Set

* Only vaccinated Schoolgirls were enrolled at Year-4 survey

**Supplementary Figure 5: Human Papillomavirus (HPV) 31, 33, and 45 Antibody Titers in Grade 8 Schoolgirls at Baseline, Grade 10 Schoolgirls in Two-year Post-Vaccination, and Grade 12 Schoolgirls in Four-year Post-Vaccination**

**
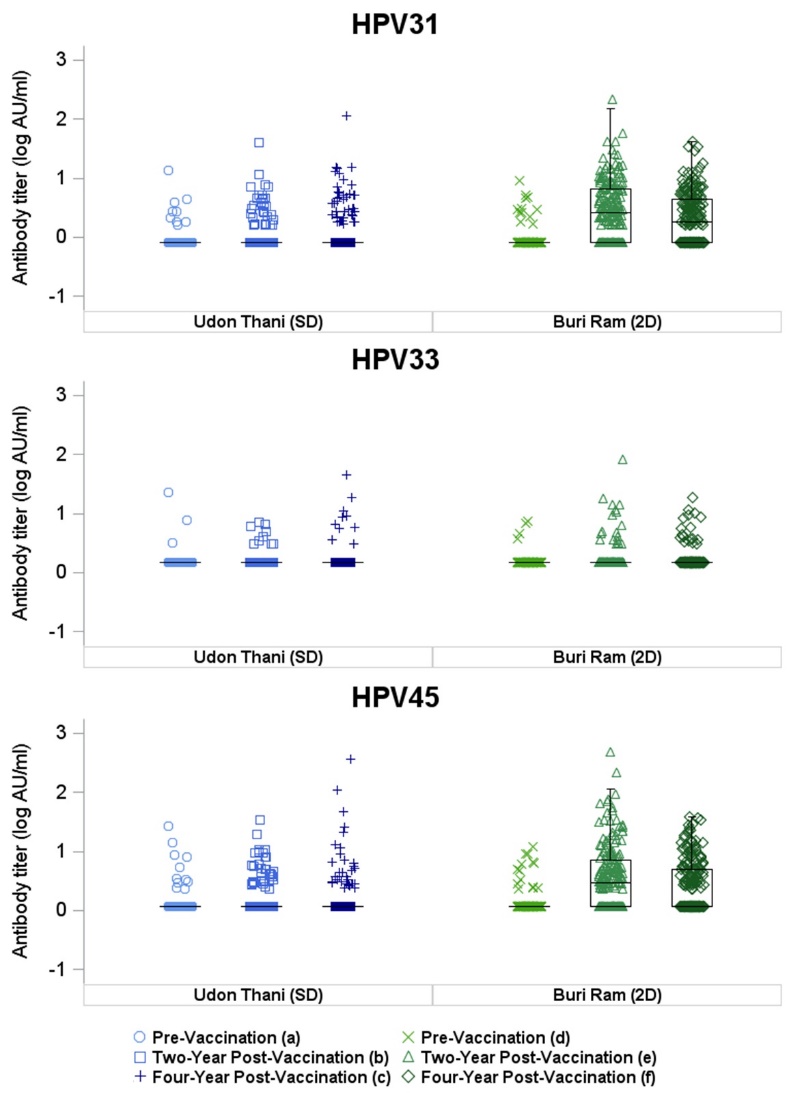
**

SD: Single-Dose, 2D: Two-Dose

Results for HPV genotypes are reported in Abitrary Unit (AU). Samples with undetectable titers were replaced by multiplying 0.5 to type-specific lower limit of quantitation.

a,d Grade 8 Schoolgirls prior to HPV vaccination campaign in Udon Thani (a) and Buri Ram (d)

b,e Two-Year Post-Vaccination among General High School Grade 10 and Vocational School Year-1 in Udon Thani (b) and Buri Ram (e)

c,f Four-Year Post-Vaccination among General High School Grade 12 and Vocational School Year-3 in Udon Thani (c) and Buri Ram (f)

**Appendix 1**

We considered three methods for calculating adjusted estimates of VE. These methods attempt to account for differences in sexual activity and risk for HPV acquisition. As sexual activity is not directly measured, these methods such as the prevalence of the 7 non-vaccine high-risk HPV genotypes (35, 39, 56, 58, 59, 66, 68) without consistent evidence of vaccine cross protection are a surrogate. The test results for these genotypes will be obtained from the combination of Cobas and Anyplex test, i.e., the Anyplex test results for the 7 genotypes will be used when available and, if not available, the test results will be set to “negative”.

**Method 1**

This method considers the 7 non-vaccine HPV genotypes. Let $p_{1}$ and $p_{2}$ denote the weighted HPV 16/18 prevalence rates in the baseline and post-vaccination survey group, respectively. Similarly, let $q_{1}$ and $q_{2}$ denote the weighted overall prevalence rates^*^ for the non-vaccine genotypes in the baseline and post-vaccination survey group, respectively. The overall prevalence rate for the non-vaccine genotypes is defined in this case as the average of the 7 genotype-specific weighted prevalence rates. The adjusted VE estimate is given by

$$1-\frac{p_{2}/q_{2}}{p_{1}/q_{1}}.$$

A 95% CI for this estimate will be constructed using a delta method.

^*^ Prevalence rate is calculated by total number of infections by non-targeted type/total number of subjects

**Method 2**

This method accounts for potential changes in the prevalence of the 7 non-vaccine genotypes as well as differences in the distribution of student’s age between baseline and post-vaccination survey group. Stratified versions of the prevalence rates will be computed with the stratification based on appropriately defined propensity scores. The propensity scores will be computed as the probability of membership in the post-vaccination survey group estimated from the following logistic regression model:

$P\left( Z=1 \right)=\frac{1}{1+exp({-a}_{0}-\sum_{i=1}^{7} {a_{i}X}_{i}-a_{8}Y)}$,

where $Z=1$ indicates that the student belongs to the post-vaccination survey group and $Z=0$ indicates the baseline group, $X_{1},...,X_{7}$ are the binary test results for the 7 genotypes and $Y$ is the student’s age. The adjusted prevalence rates for the baseline and post-vaccination survey group by stratifying the data into quintiles based on the propensity scores, a technique referred to as stratified propensity score approach. Note that the propensity-adjusted prevalence rates will be computed separately for the two school types and the overall estimate will be obtained as a weighted average to account for oversampling of vocational schools. The propensity-adjusted vaccine effectiveness estimates will be computed from the resulting prevalence rates and 95% CIs will be obtained using the delta method.

**Method 3**

This method accounts for potential changes in the proportion of self-reported sexual activity between the baseline and post-vaccination groups. Let $p_{1}$ and $p_{2}$ denote the weighted HPV 16/18 prevalence rates in the baseline and post-vaccination group, respectively. The $q_{1}$ and $q_{2}$ denote the weighted proportion of individuals with sexual activity in the baseline and post-vaccination groups, respectively. For the proportion of sexual activity, a total number of schoolgirls who responded "yes" to "Have you ever had sex?" question will be the numerator and a total number of schoolgirls who responded to the sexual behavioral questionnaire will be the denominator. The adjusted VE estimate is given by

$$1-\frac{p_{2}/q_{2}}{p_{1}/q_{1}}.$$

A 95% CI for this estimate will be constructed using a delta method.

Vaccine effectiveness (VE) for cross-protective types will be also estimated using the above three methods.
